# Supplementary material for: Conjugation of Palbociclib with MHI-148 Has an Increased Cytotoxic Effect for Breast Cancer Cells and an Altered Mechanism of Action
Source: Molecules. 2022 Jan 27;27(3):880. doi: 10.3390/molecules27030880 (PMC8840619; doi:10.3390/molecules27030880)
Supplement: Supplementary file 1 [file molecules-27-00880-s001.zip › molecules-1502955-supplementary.pdf]

Supplementary Materials

Conjugation of Palbociclib with MHI-148 has an Increased Cytotoxic Effect for Breast Cancer Cells and an Altered Mechanism of Action

Peter Jaein Choi [1,2], Petr Tomek [1], Moana Tercel [1,2], Jóhannes Reynisson [3], Thomas In Hyeup Park [4], Elizabeth Alexandra Cooper [4], William Alexander Denny [1,2], Jiney Jose [1,2,*] and Euphemia Leung [1,2,*]

[1] Auckland Cancer Society Research Centre, Faculty of Medical and Health Sciences, University of Auckland, Auckland 1023, New Zealand; p.choi@auckland.ac.nz (P.J.C.); p.tomek@auckland.ac.nz (P.T.); m.tercel@auckland.ac.nz (M.T.); b.denny@auckland.ac.nz (W.A.D.)#
[2] Maurice Wilkins Centre for Molecular Biodiscovery, University of Auckland, Auckland 1023, New Zealand
[3] School of Pharmacy and Bioengineering, Keele University, Hornbeam Building, Newcastle under Lyme, ST5 5BG Staffordshire, UK; j.reynisson@keele.ac.uk
[4] Neurosurgical Research Unit & Department of Pharmacology, The Centre for Brain Research, University of Auckland, Private Bag 92019, Auckland 1023, New Zealand; Thomas.park@auckland.ac.nz (T.I.H.P.); e.cooper@auckland.ac.nz (E.A.C.)
* Correspondence: j.jose@auckland.ac.nz (J.J.); e.leung@auckland.ac.nz (E.L.)#

Supplementary Table S1. EC_{50} values of palbociclib, MHI-148 and MHI-palbociclib in breast cancer MCF-7 and MDA-MB-231 cell lines, and non-cancerous HEK293, 51D1 and 51D1.3 cell lines using WST-1 assay.

		**MCF-7**	**MDA-MB-231**	**HEK293**	**51D1**	**51D1.3**
**EC50 (nM) WST-1 assay**	**Palbociclib**	>2500	>2500	>2500	>2500	>2500
	**MHI-148**	>2500	>2500	>2500	>2500	>2500
	**MHI-palbociclib**	718.8 ± 74.1	871.6 ± 98.9	543.8 ± 5.9	265.0 ± 20.3	471.3 ± 61.2
